# Pregnancy and neonatal outcomes in women with arcuate uterus: a population-based cohort study of over 3.8 million women

**DOI:** 10.1186/s12884-025-08125-7

**Published:** 2025-10-28

**Authors:** Nir Kugelman, Ella M. Gangbe, Ahmad Badeghiesh, Haitham Baghlaf, Michael H. Dahan

**Affiliations:** 1 Rappaport Faculty of Medicine, Technion Israel Institute of Technology, Technion City, Haifa 3200003 Israel; 2 Department of Obstetrics and Gynecology, Division of Reproductive Endocrinology and Infertility, McGill University, Montreal, Canada; 3 Department of Obstetrics and Gynecology, McGill University, Montreal, Canada; 4 Department of Obstetrics and Gynecology, King Abdulaziz University, Rabigh, Saudi Arabia; 5 Department of Obstetrics and Gynecology, University of Tabuk, Tabuk, Saudi Arabia

**Keywords:** Arcuate uterus, Cohort studies, Pregnancy complications, Pregnancy outcome, Uterine anomalies

## Abstract

**Background:**

Congenital uterine anomalies are associated with adverse reproductive outcomes, yet the impact of the arcuate uterus remains unclear due to limited sample sizes and inconsistent findings in previous studies. We utilized a large population database to assess pregnancy, delivery, and neonatal outcomes in women with an arcuate uterus.

**Methods:**

Retrospective population-based study using data from the Health Care Cost and Utilization Project-Nationwide Inpatient Sample. Cases of arcuate uterus were identified using ICD code 752.36. Pregnancies in women with an arcuate uterus were matched to 3,016 pregnancies in women without congenital uterine anomalies (1 to 4) and compared to the entire population without congenital anomalies. Multivariate logistic regression adjusted for confounding variables.

**Results:**

Among 3,841,147 control births and 754 births in women with an arcuate uterus, more of these women were older than 25 and had higher rates of previous cesarean sections (CS), in-vitro pregnancies, and multiple gestations (all *P* < 0.01). Pregnancy outcomes showed higher rates of pregnancy-induced hypertension (adjusted odds ratio (aOR) 1.32), preeclampsia (aOR 1.63), premature preterm rupture of membranes (aOR 2.86), preterm delivery (aOR 1.86), placental abruption (aOR 3.08), CS (aOR 10.88), and small for gestational age (SGA) neonates (aOR 2.21) (all 95%CI excluding 1).

Compared to the matched cohort, women with an arcuate uterus had higher rates of pregnancy-induced hypertension (aOR 1.76), preeclampsia (aOR 2.08), premature preterm rupture of membranes (aOR 2.46), preterm delivery (aOR 2.74), placental abruption (aOR 2.11), postpartum hemorrhage (aOR 1.67), wound complications (aOR 3.42), CS (aOR 30.04), disseminated intravascular coagulopathy (DIC) (aOR 5.36), and SGA (aOR 1.76) (all 95%CI excluding 1).

**Conclusions:**

Women with an arcuate uterus appear to be at increased risk of adverse pregnancy outcomes, particularly CS and DIC. These associations should be interpreted with caution and confirmed in larger studies.

## Background

Congenital uterine anomalies (CUAs) affect about 5% of women and are linked to adverse health outcomes [1]. In non-pregnant women, CUAs can cause hematometra, hematocolpos, endometriosis, pelvic pain, abnormal uterine bleeding, and genital and urinary tract infections [2]. Additionally, 20–30% of individuals with Müllerian anomalies present with renal anomalies and other malformations, including cardiac defects [3–5].

In pregnant women, CUAs increase the risk of first and second-trimester miscarriages, preterm delivery, malpresentation, cesarean section (CS), and small for gestational age (SGA) neonates [6, 7]. Women with infertility also show higher rates of uterine malformations [8]. These adverse outcomes are primarily associated with uterus didelphys, bicornuate uterus, and septate uterus [3].

The arcuate uterus, a common CUA, is characterized by a slight indentation of less than a centimeter at the fundus with an angle above 90 degrees [9]. Most women with an arcuate uterus are asymptomatic, often diagnosed incidentally during ultrasonography [10]. Compared to other CUAs, the arcuate uterus appears to have fewer adverse effects on pregnancies. For instance, Woelfer et al. reported a slight increase in second-trimester pregnancy losses and preterm labor among women with an arcuate uterus [11], while Mucowski et al. found no significant impact on reproductive outcomes in a smaller study [12].

Most literature focuses on more significant uterine anomalies such as bicornuate or unicornuate uteri since arcuate uterus is often considered a variation of normal [12] or combines all CUAs. Studies specifically examining the effects of the arcuate uterus on pregnancy outcomes involved only a small number of participants, potentially lacking the power to detect significant differences. The largest study on this topic examined 420 cases of arcuate uterus and small septum combined [13]. Additionally, findings in the literature are conflicting. This study aims to clarify the pregnancy, delivery, and neonatal outcomes of women with an arcuate uterus using a large population database.

## Methods

### Data source

We conducted a retrospective population-based cohort study using data from the Health Care Cost and Utilization Project-Nationwide Inpatient Sample (HCUP-NIS) from 2010 to 2014. The HCUP-NIS database contains data on hospital inpatient stays and represents the largest publicly available all-payer inpatient database in the United States, covering over 97% of inpatient discharges from U.S. community hospitals. Data collection is managed by federal and state agencies, hospital associations, and private organizations. Data elements before 2015 are coded using the International Classification of Diseases, Ninth Revision, Clinical Modification (ICD-9-CM).

### Study population

In-hospital births occurring between 2010 and 2014 were included in the study. Births were identified using ICD-9-CM diagnostic codes (634x-679x, V22x, V23x, V27x) and procedural codes (72x-75x). Each pregnancy was included only once. In-hospital admissions resulting in delivery or maternal death were identified using specific ICD-9-CM codes (650x, 677x, 651x-676x) and procedural codes (72x, 73x, 74.0, 74.1, 74.2, 74.4, 74.99). Women with an arcuate uterus were identified using ICD-9-CM diagnostic code 752.36. Women without uterine anomalies served as the control group.

### Data collection

Baseline characteristics recorded included age, race, type of insurance, income, body mass index (BMI) (obese BMI ≥ 30 kg/m² versus non-obese BMI < 30 kg/m²), previous CS, smoking history, pre-existing hypertension, diabetes, thyroid disease, drug use, HIV status, use of in in vitro fertilization (IVF), and presence of multiple gestation.

Pregnancy outcomes assessed included pregnancy-induced hypertension (PIH), chronic hypertension with superimposed preeclampsia, gestational hypertension, preeclampsia, eclampsia, gestational diabetes, and placenta previa. In the ICD-9-CM system, PIH was recorded as a composite category reflecting older terminology, which encompassed hypertensive disorders such as gestational hypertension, preeclampsia, and eclampsia. These outcomes were also analyzed separately when specifically coded.

Delivery outcomes included preterm premature rupture of membranes (PPROM), preterm delivery, placental abruption, chorioamnionitis, mode of delivery (operative vaginal delivery, CS, spontaneous vaginal delivery (SVD)), hysterectomy, postpartum hemorrhage (PPH), wound complications, maternal death, blood transfusion, maternal infection, venous thromboembolism (VTE), and disseminated intravascular coagulopathy (DIC). Deep vein thrombosis (DVT) and pulmonary embolism (PE) were included under VTE. Neonatal outcomes included SGA, intrauterine fetal demise, and congenital anomalies.

### Statistical analysis

An unmatched and a matched analysis were performed. Each case of arcuate uterus was matched to four controls based on age, race, income, and insurance type. Statistically significant differences in baseline characteristics between women with and without an arcuate uterus were compared using Chi-Squared or Fisher’s exact tests. Maternal and neonatal outcomes were compared using univariate and multivariate logistic regression analyses adjusted for confounding variables (characteristics with *p* < 0.05). Analyses were conducted using SPSS 23.0 software. Statistical significance was set at *p* < 0.05.

## Results

**Fig. 1:**
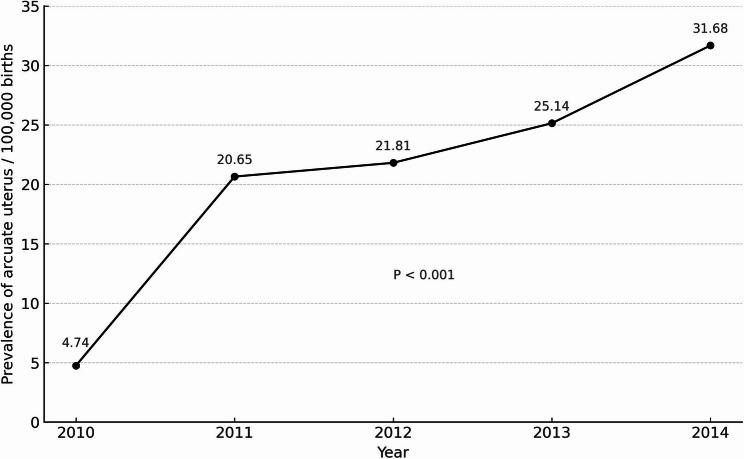
Prevalence of arcuate uterus among the 3,840,901 births that occurred between 2010 and 2014

A total of 3,851,029 births occurred during the study period, including 754 from women with an arcuate uterus and 3,840,147 from women without CUAs (control group). Additionally, 10,128 births from women with other CUAs were excluded. The 754 births from women with an arcuate uterus were matched to 3,016 control births. The prevalence of arcuate uterus between 2010 and 2014 was 19.58 per 100,000 births, with a significant increase over time from 4.74 per 100,000 in 2010 to 31.68 per 100,000 in 2014 (*p* < 0.001) (Fig. 1).

### Baseline characteristics

Women with an arcuate uterus were more likely to be older than 25 years, Caucasian, in a higher income quartile, and have private insurance compared to the control group (all *p* < 0.001) (Table 1). They were also more likely to have had a previous CS (*p* < 0.001), pre-gestational diabetes (*p* = 0.03), thyroid disease (*p* < 0.001), IVF pregnancies (*p* < 0.01), and multiple gestations (*p* < 0.01). In the matched group, women with an arcuate uterus were more likely to have had a previous CS (*p* < 0.001) and thyroid disease (*p* = 0.02).

**Table 1:** Maternal characteristics of the 3,840,901 births that occurred between 2004 to 2014

Characteristics	Arcuate Uterus *N* = 754	No Arcuate Uterus *N* = 3,840,147	*p*-value
Age (years)			< 0.001
< 25	182 (24.1%)	1,379,779 (35.8%)	
25–34	443 (58.7%)	1,894,732 (49.3%)	
≥ 35	128 (17.0%)	572,637 (14.9%)	
Race			< 0.001
White	534 (70.8%)	2,036,437 (53.0%)	
Black	29 (3.8%)	560,659 (14.6%)	
Hispanic	117 (15.5%)	825,628 (21.5%)	
Asian and Pacific	42 (5.6%)	20,736 (5.4%)	
Native American	5 (0.7%	30,721 (0.8%)	
Other	28 (3.7%)	184,327 (4.8%)	
Income quartiles			< 0.001
Less than 39,000	118 (15.6%)	1,063,521 (27.7%)	
$39,000–47,999	167 (22.2%)	960,037 (25.0%)	
$48,000–62,999	224 (29.7%)	967,705 (25.2%)	
$63,000 or more	245 (32.5%)	848,589 (22.1%)	
Plan type			< 0.001
Medicare	8 (1.1%)	26,881 (0.7%)	
Medicaid	191 (25.3%)	1,686,081 (43.9%)	
Private including Health Maintenance Organizations	511 (67.8%)	1,911,655 (49.8%)	
Self-pay	18 (2.4%)	99,844 (2.6%)	
No charge	0 (0.0%)	3840 (0.1%)	
Other	26 (3.4%)	111,364 (2.9%)	
Obesity	46 (6.1%)	211,207 (5.5%)	0.50
Previous cesarean section	194 (25.7%)	656,486 (17.1%)	< 0.001
Smoking during pregnancy	41 (5.4%)	211,207 (5.5%)	0.98
Chronic hypertension	16 (2.1%)	84,483 (2.2%)	0.93
Pregestational diabetes	13 (1.7%)	38,401 (1.0%)	0.03
Drug use	14 (1.9%)	61,442 (1.6%)	0.64
Thyroid disease	48 (6.4%)	119,045 (3.1%)	< 0.001
HIV	0 (0.0%)	0 (0.0%)	1.00
In vitro fertilization	6 (0.8%)	7680 (0.2%)	< 0.01
Multiple gestation	25 (3.3%)	65,282 (1.7%)	< 0.01

*HIV* Human immunodeficiency virus

### Pregnancy outcomes

Pregnancy outcomes in both matched and unmatched groups showed that women with an arcuate uterus had higher risks of PIH (adjusted odd ratio (aOR) 1.76, 95% CI 1.34–2.32 in the matched group; aOR 1.32, 95% CI 1.03–1.70 in the unmatched group) and preeclampsia (aOR 2.08, 95% CI 1.45–2.98 in the matched group; aOR 1.63, 95% CI 1.18–2.24 in the unmatched group) (Table 2). However, they were not at higher risk for gestational hypertension, eclampsia, chronic hypertension with superimposed preeclampsia, gestational diabetes, or placenta previa.

**Table 2:** Pregnancy, delivery and other outcomes in women with an arcuate uterus compared to matched controls

Outcomes	Arcuate uterus *n* = 754	No Arcuate Uterus *n* = 3,840,147	Crude Odds Ratio (95% Confidence Interval)	Adjusted Odds Ratio (95% Confidence Interval)	Adjusted *p*-value
Pregnancy outcomes^†^					
Pregnancy induced HTN	82 (10.9%)	307,212 (8.0%)	1.40 (1.11–1.76)	1.32 (1.03–1.70)	0.03
Gestational HTN	30 (4.0%)	141,085 (3.7%)	1.09 (0.76–1.58)	1.02 (0.70–1.51)	0.92
Preeclampsia	48 (6.4%)	145,925 (3.8%)	1.72 (1.29–2.31)	1.63 (1.18–2.24)	< 0.01
Eclampsia	1 (0.1%)	3840 (0.1%)	1.94 (0.27–13.8)	2.55 (0.36–18.12)	0.35
Preeclampsia and Eclampsia superimposed HTN	6 (0.8%)	23,041 (0.6%)	1.25 (0.56–2.80)	1.22 (0.51–2.96)	0.66
GDM	57 (7.6%)	249,609 (6.5%)	1.18 (0.90–1.55)	1.07 (0.80–1.43)	0.66
Placenta previa	6 (0.8%)	23,041 (0.6%)	1.40 (0.63–3.12)	1.11 (0.46–2.69)	0.81
Delivery outcomes^‡^					
PPROM	25 (3.3%)	42,242 (1.1%)	2.96 (1.99–4.41)	2.86 (1.86–4.40)	< 0.001
Preterm delivery	90 (11.9%)	253,448 (6.6%)	1.92 (1.54–2.40)	1.86 (1.45–2.37)	< 0.001
Abruptio placenta	22 (2.9%)	42,242 (1.1%)	2.81 (1.84–4.29)	3.08 (1.99–4.77)	< 0.001
Chorioamnionitis	16 (2.1%)	72,963 (1.9%)	1.11 (0.67–1.81)	1.21 (0.71–2.06)	0.48
Operative vaginal delivery	16 (1.2%)	180,285 (4.7%)	0.25 (0.13–0.47)	0.26 (0.13–0.52)	< 0.001
Cesarean section	612 (81.2%)	1,255,948 (32.7%)	8.82 (7.35–10.59)	10.88 (8.90–13.30)	< 0.001
Spontaneous vaginal delivery	133 (17.6%)	2,395,092 (62.4%)	0.13 (0.11–0.16)	0.12 (0.10–0.14)	< 0.001
Hysterectomy	0 (0.0%)	3840 (0.1%)	-	-	-
PPH	32 (4.2%)	115,204 (3.0%)	1.42 (0.99–2.02)	1.43 (0.99–2.08)	0.06
Wound complications	7 (0.9%)	11,520 (0.3%)	2.81 (1.34–5.92)	2.14 (0.95–4.78)	0.07
Maternal death	0 (0.0%)	0 (0.0%)	-	-	-
Transfusion	14 (1.9%)	46,082 (1.2%)	1.56 (0.92–2.65)	1.50 (0.84–2.66)	0.17
Others					
Maternal infection	18 (2.4%)	88,323 (2.3%)	1.05 (0.66–1.68)	1.18 (0.72–1.94)	0.52
DVT	0 (0.0%)	0 (0.0%)	-	-	-
Pulmonary embolism	2 (0.3%)	0 (0.0%)	13.58 (3.38–54.49)	15.14 (3.76–60.91)	< 0.001
VTE	2 (0.3%)	3840 (0.1%)	4.61 (1.15–18.50)	4.81 (1.20–19.30)	0.03
DIC	4 (0.5%)	7680 (0.2%)	2.17 (0.81–5.80)	2.19 (0.82–5.88)	0.12

*HTN* hypertension, *GDM* gestational diabetes mellitus, *PPROM* premature rupture of membranes, *PPH* post-partum hemorrhage, *DVT* deep vein thrombosis, *VTE* venous thromboembolism, *DIC* disseminated intravascular coagulation

^†^ Pregnancy outcomes: Adjusted for Age, Race, Plan types, Income quartiles, Previous cesarean section, Thyroid disease, Multiple gestation, Pregestational diabetes and IVF

^‡^ Delivery outcomes: Adjusted for Age, Race, Plan types, Income quartiles, Previous cesarean section, Thyroid disease, Multiple gestation, Pregestational diabetes, IVF, Pregnancy induce hypertension and Preeclampsia

### Delivery outcomes

Women with an arcuate uterus were more likely to experience PPROM (aOR 2.46, 95% CI 1.48–4.09 in the matched group; aOR 2.86, 95% CI 1.86–4.40 in the unmatched group), preterm delivery (aOR 2.74, 95% CI 2.06–3.65 in the matched group; aOR 1.86, 95% CI 1.45–2.37 in the unmatched group), and placental abruption (aOR 2.11, 95% CI 1.23–3.63 in the matched group; aOR 3.08, 95% CI 1.99–4.77 in the unmatched group) (Table 2). They also had significantly higher odds of delivering via CS (aOR 30.04, 95% CI 23.99–37.63 in the matched group; aOR 10.88, 95% CI 8.90–13.30 in the unmatched group) and were much less likely to have a spontaneous vaginal delivery (SVD) (aOR 0.04, 95% CI 0.03–0.05 in the matched group; aOR 0.12, 95% CI 0.10–0.14 in the unmatched group).

In the matched cohort, women with an arcuate uterus had higher odds of PPH (aOR 1.67, 95% CI 1.09–2.56), wound complications (aOR 3.42, 95% CI 1.18–9.96), and DIC (aOR 5.58, 95% CI 1.21–25.60). In the unmatched group, they had higher odds of VTE (aOR 4.81, 95% CI 1.20–19.30) PE (aOR 15.14, 95% CI 3.76–60.91). No maternal deaths were reported in either group, and no significant differences were found in other reported outcomes.

### Neonatal outcomes

Deliveries from women with an arcuate uterus had a 5.7% rate of SGA neonates compared to 3.2% in the matched control group (aOR 1.76, 95% CI 1.21–2.56) and 2.6% in the unmatched control group (aOR 2.21, 95% CI 1.58–3.10) (Table 3). Rates of intrauterine fetal demise and congenital anomalies were similar between the groups in both the matched and unmatched cohorts.

**Table 3:** Neonatal outcomes in women with an arcuate uterus compared to matched controls

Outcomes^†^	Arcuate uterus *N* = 754	No Arcuate Uterus *N* = 3,840,147	Crude Odds Ratio (95% Confidence Interval)	Adjusted Odds Ratio (95% Confidence Interval)	Adjusted *p*-value
Small for gestational age	43 (5.7%)	99,844 (2.6%)	2.30 (1.69–3.13)	2.21 (1.58–3.10)	< 0.001
Intra uterine fetal demise	5 (0.7%)	15,361 (0.4%)	1.59 (0.66–3.83)	1.97 (0.82–4.75)	0.13
Congenital anomalies	0 (0.0%)	11,520 (0.3%)	-	-	-

^†^Neonatal outcomes: Adjusted for Age, Race, Plan type, Income quartiles, Previous cesarean section, Thyroid Disease, Multiple gestation, Pregestational diabetes, IVF, Pregnancy induced hypertension and Preeclampsia

## Discussion

This large-scale, population-based study is the first to evaluate the impact of an arcuate uterus on obstetrical and neonatal outcomes using a substantial cohort. Our findings indicate that women with an arcuate uterus have higher risks of several complications, including PIH, preeclampsia, preterm delivery, PPROM, placental abruption, CS, PPH, and wound complications. Despite these increased risks, rates of intrauterine fetal demise and congenital anomalies were similar between women with and without an arcuate uterus. Additionally, deliveries in women with an arcuate uterus showed higher odds of SGA neonates.

The arcuate uterus is one of the most common CUAs. The reported prevalence varies depending on the population studied and the mode of diagnosis. In a systematic review, Chan et al. demonstrated a prevalence of 3.9% of arcuate uterus in an unselected population [1]. In studies estimating the prevalence of CUAs in women with fertility issues, the arcuate uterus was found in 12–16% of that specific population [14–16]. Our results demonstrate a prevalence of an arcuate uterus of 19.58 per 100,000 births between 2010 and 2014. This prevalence is substantially lower than that reported in clinical studies, which likely reflects the limited sensitivity of ICD-9-CM coding in capturing this anomaly. Because coding is applied only when a definitive diagnosis is documented, the specificity is high, but many cases are likely missed, resulting in underestimation. Importantly, the dramatic six-fold increase in prevalence observed over the study years cannot be attributed to any change in ICD-9-CM diagnostic coding, which remained constant during this period. Instead, this rise most likely reflects improved recognition, documentation, and diagnostic imaging practices. Taken together, these factors suggest that our estimates may underestimate the true prevalence and risks associated with arcuate uterus in pregnancy.

Women with an arcuate uterus had a higher incidence of hypertensive disorders of pregnancy (including pregnancy-induced hypertension and preeclampsia). Not many studies have specifically looked at this outcome in CUAs. Fox et al. described the possibility of unilateral placental implantation and functional exclusion of one of the uterine arteries as potential causes for increased rates of intrauterine growth restriction and preeclampsia in women with major uterine anomalies. In their study, the arcuate uterus did not seem to be associated with increased preeclampsia; however, they had only 14 cases with the malformation, which might not have provided enough power to detect the risk of preeclampsia [6]. It is thus unclear if the arcuate uterus could also lead to abnormal placentation and possible hypertensive disorders during pregnancy.

As demonstrated by others, the arcuate uterus was also associated with thirty times the rates of CS delivery and lower rates of spontaneous vaginal delivery. This could be explained by the fact that malpresentations and placental abruption are more common [17, 18]. Thus, it is not surprising that these women were more likely to have had CS in the past. Higher rates of CS and the associated coagulopathy, as well as the possible higher risk of hypertensive disorders in pregnancy, could directly explain why women with an arcuate uterus had a higher risk of postpartum hemorrhage and five times more DIC. This increase in the risk of postpartum hemorrhage and DIC was only found in the matched cohort. In the unmatched cohort, women with an arcuate uterus had twelve times more PE and five times more deep vein thrombosis events. This difference was not found once the cases were matched to controls. The increased risk of DIC noted in our results might not, however, be significant, as only a very small number of women suffered from these outcomes. It is unlikely that the arcuate uterus itself would cause an increased risk of coagulability.

In our study, women with an arcuate uterus demonstrated about twice the risk for PPROM, preterm delivery, and SGA. Most of the published literature recognizes these risks with CUAs as a whole but not necessarily with the arcuate uterus [13, 19, 20]. Lekovich et al. suggested placental malperfusion as a possible cause of preterm delivery in women with uterine anomalies. Placental malperfusion is also a recognized cause of small neonates [18]. Woelfer et al. and Fox et al. found that women with an arcuate uterus had higher rates of second-trimester miscarriage and preterm delivery compared to those with a normal uterus. Their respective study populations included 72 and 14 women with the anomaly [6, 11]. However, a systematic review of 12 articles looking partly at the outcomes of the arcuate uterus did not find any of these differences, although the relative risk was marginally insignificant at 2.04 (95% CI 0.99 to 4.1) [21]. Our larger cohort might have provided sufficient power to confirm that the arcuate uterus is indeed associated with risks of PPROM, preterm delivery, and SGA.

Whether metroplasty of the arcuate uterus improves pregnancy outcomes is controversial. One prospective study of 96 women with arcuate and septate uteri found no difference in outcomes [22]. Conversely, a larger retrospective study of 420 small septums and arcuate uteri reported a decrease in the rate of preterm birth from 34% before hysteroscopic resection to 7% after, and a decrease in the rate of extreme preterm birth from 13 to 3% [13].

A recent case-control study also looked at the pregnancy outcomes of 37 women with an arcuate uterus compared to 165 women with similar baseline characteristics and no congenital uterine anomalies. Similar to our study, their results showed that women with an arcuate uterus were at increased risk of spontaneous preterm birth and had lower mean birthweight. However, there were no differences in the risks of preeclampsia or CS [23].

The study has several limitations. First, its retrospective design and reliance on ICD-9-CM coding for identifying cases may have introduced classification bias. In particular, some women with a low-level septate uterus may have been misclassified as having an arcuate uterus. Second, the database lacks detailed clinical data on the method of diagnosis, parity, fertility history, previous pregnancy losses, indications for CS, history of coagulopathies, and prior adverse outcomes. The absence of these variables may confound our findings and limits our ability to determine causality. Third, the relatively low prevalence of arcuate uterus identified in the database likely reflects under-detection, which would tend to underestimate rather than exaggerate the risks associated with this anomaly. Taken together, these factors highlight that our results should be interpreted with caution and viewed as hypothesis-generating rather than definitive.

The strengths of this study include its large cohort size, making it the most extensive study to date examining pregnancy, delivery, and neonatal outcomes associated with the arcuate uterus. The study design involved matching cases of arcuate uterus to controls, and we adjusted for confounding variables using multivariate logistic regression analysis. This robust methodology enhances confidence in our findings despite the heterogeneity in the existing literature.

Additionally, even if some subjects had low-level septate uteri rather than truly arcuate uteri, the results likely reflect the complications in the population diagnosed with arcuate uteri. We expect the rate of misdiagnosis to be consistent between our study and the general population, thereby supporting the generalizability of our findings. Consequently, our results provide valuable insights that can guide practitioners in understanding and managing the pregnancy risks associated with an arcuate uterus.

## Conclusions

Women with an arcuate uterus appear to be at increased risk for several adverse pregnancy outcomes, including hypertensive disorders of pregnancy (pregnancy-induced hypertension and preeclampsia), PPROM, preterm delivery, placental abruption, CS, PPH, DIC, SGA neonates, and wound complications. These findings highlight the need for careful monitoring of this population. However, given the limitations of our data, the associations we observed should be interpreted with caution. Further large-scale prospective studies are warranted to confirm these findings and to guide evidence-based interventions.

## Data Availability

The dataset supporting the conclusions of this article is available in the Health Care Cost and Utilization Project-Nationwide Inpatient Sample (HCUP-NIS) repository, https://health.gov/healthypeople/objectives-and-data/data-sources-and-methods/data-sources/healthcare-cost-and-utilization-project-national-nationwide-inpatient-sample-hcup-nis.

## Abbreviations

CUAs: Congenital uterine anomalies
CS: Cesarean section
SGA: Small for gestational age
HCUP-NIS: Project-Nationwide Inpatient Sample
ICD-9-CM: International Classification of Diseases, Ninth Revision, Clinical Modification
BMI: Body mass index
IVF: In vitro fertilization
PIH: Pregnancy-induced hypertension
PPROM: Preterm premature rupture of membranes
SVD: Spontaneous vaginal delivery
PPH: Postpartum hemorrhage
VTE: Venous thromboembolism
DIC: Disseminated intravascular coagulopathy
DVT: Deep vein thrombosis
PE: Pulmonary embolism
aOR: Adjusted odd ratio
CI: Confidence interval

## References

[1] Chan YY, Jayaprakasan K, Zamora J, Thornton JG, Raine-Fenning N, Coomarasamy A. The prevalence of congenital uterine anomalies in unselected and high-risk populations: a systematic review. Hum Reprod Update. 2011;17:761–71. 21705770 10.1093/humupd/dmr028 PMC3191936

[2] Management of acute obstructive uterovaginal anomalies. ACOG committee opinion, number 779. Obstet Gynecol. 2019;133:e363–71. 31135762 10.1097/AOG.0000000000003281

[3] Lin PC, Bhatnagar KP, Nettleton GS, Nakajima ST. Female genital anomalies affecting reproduction. Fertil Steril. 2002;78:899–915. 12413972 10.1016/s0015-0282(02)03368-x

[4] Oppelt P, von Have M, Paulsen M, Strissel PL, Strick R, Brucker S, et al. Female genital malformations and their associated abnormalities. Fertil Steril. 2007;87:335–42. 17126338 10.1016/j.fertnstert.2006.07.1501

[5] Pittock ST, Babovic-Vuksanovic D, Lteif A. Mayer-Rokitansky-Küster-Hauser anomaly and its associated malformations. Am J Med Genet A. 2005;135:314–6. 15887261 10.1002/ajmg.a.30721

[6] Fox NS, Roman AS, Stern EM, Gerber RS, Saltzman DH, Rebarber A. Type of congenital uterine anomaly and adverse pregnancy outcomes. J Matern Fetal Neonatal Med. 2014;27:949–53. 24050215 10.3109/14767058.2013.847082

[7] Hua M, Odibo AO, Longman RE, Macones GA, Roehl KA, Cahill AG. Congenital uterine anomalies and adverse pregnancy outcomes. Am J Obstet Gynecol. 2011;205:e5581–5. 10.1016/j.ajog.2011.07.022 21907963

[8] Hassan M-AM, Lavery SA, Trew GH. Congenital uterine anomalies and their impact on fertility. Womens Health (Lond Engl). 2010;6:443–61. 20426609 10.2217/whe.10.19

[9] Ludwin A, Martins WP, Nastri CO, Ludwin I, Coelho Neto MA, Leitão VM, et al. Congenital uterine malformation by experts (CUME): better criteria for distinguishing between normal/arcuate and septate uterus? Ultrasound Obstet Gynecol. 2018;51(1):101–9. 29024135 10.1002/uog.18923

[10] Jayaprakasan K, Ojha K. Diagnosis of congenital uterine abnormalities: practical considerations. J Clin Med. 2022;11(5):1251. 10.3390/jcm11051251 PMC8911320 35268343

[11] Woelfer B, Salim R, Banerjee S, Elson J, Regan L, Jurkovic D. Reproductive outcomes in women with congenital uterine anomalies detected by three-dimensional ultrasound screening. Obstet Gynecol. 2001;98(6):1099–103. 11755560 10.1016/s0029-7844(01)01599-x

[12] Mucowski SJ, Herndon CN, Rosen MP. The arcuate uterine anomaly: a critical appraisal of its diagnostic and clinical relevance. Obstet Gynecol Surv. 2010;65(7):449–54. 20723266 10.1097/OGX.0b013e3181efb0db

[13] Tomazevic T, Ban-Frangez H, Ribic-Pucelj M, Premru-Srsen T, Verdenik I. Small uterine septum is an important risk variable for preterm birth. Eur J Obstet Gynecol Reprod Biol. 2007;135(2):154–7. 17182166 10.1016/j.ejogrb.2006.12.001

[14] Zhang Y, Zhao Y, Qiao J. Obstetric outcome of women with uterine anomalies in China. Chin Med J. 2010;123(4):418–22. 20193480

[15] Jayaprakasan K, Chan YY, Sur S, Deb S, Clewes JS, Raine-Fenning NJ. Prevalence of uterine anomalies and their impact on early pregnancy in women conceiving after assisted reproduction treatment. Ultrasound Obstet Gynecol. 2011;37(6):727–32. 21337662 10.1002/uog.8968

[16] Prior M, Richardson A, Asif S, Polanski L, Parris-Larkin M, Chandler J, et al. Outcome of assisted reproduction in women with congenital uterine anomalies: a prospective observational study. Ultrasound Obstet Gynecol. 2018;51(1):110–7. 29055072 10.1002/uog.18935

[17] Żyła MM, Wilczyński J, Nowakowska-Głąb A, Maniecka-Bryła I, Nowakowska D. Pregnancy and delivery in women with uterine malformations. Adv Clin Exp Med. 2015;24(5):873–9. 26768640 10.17219/acem/23171

[18] Lekovich J, Stewart J, Anderson S, Niemasik E, Pereira N, Chasen S. Placental malperfusion as a possible mechanism of preterm birth in patients with müllerian anomalies. J Perinat Med. 2017;45(1):45–9. 27639266 10.1515/jpm-2016-0075

[19] Saravelos SH, Cocksedge KA, Li T-C. The pattern of pregnancy loss in women with congenital uterine anomalies and recurrent miscarriage. Reprod Biomed Online. 2010;20(3):416–22. 20093084 10.1016/j.rbmo.2009.11.021

[20] Carbonnel M, Pirtea P, de Ziegler D, Ayoubi JM. Uterine factors in recurrent pregnancy losses. Fertil Steril. 2021;115(3):538–45. 33712099 10.1016/j.fertnstert.2020.12.003

[21] Venetis CA, Papadopoulos SP, Campo R, Gordts S, Tarlatzis BC, Grimbizis GF. Clinical implications of congenital uterine anomalies: a meta-analysis of comparative studies. Reprod Biomed Online. 2014;29(6):665–83. 25444500 10.1016/j.rbmo.2014.09.006

[22] Gergolet M, Campo R, Verdenik I, Kenda Suster N, Gordts S, Gianaroli L. No clinical relevance of the height of fundal indentation in subseptate or arcuate uterus: a prospective study. Reprod Biomed Online. 2012;24(5):576–82. 22417666 10.1016/j.rbmo.2012.01.025

[23] Connolly CT, Hill MB, Klahr RA, Zafman KB, Rebarber A, Fox NS. Arcuate uterus as an independent risk factor for adverse pregnancy outcomes. Am J Perinatol. 2024;41(2):167–73. 34670319 10.1055/a-1674-5927

